# Supplementary material for: TADKB: Family classification and a knowledge base of topologically associating domains
Source: BMC Genomics. 2019 Mar 14;20:217. doi: 10.1186/s12864-019-5551-2 (PMC6419456; doi:10.1186/s12864-019-5551-2)
Supplement: Supplementary file 1 — Figure S1. The 30 × 30 average Hi-C contact matrices calculated from all members in a TAD family. The family is from GM12878 with 20 predefined chromatin-state clusters and five predefined structural clusters. Figure S2. An example of two Hi-C heat maps for TAD pairs in acrossCells. Table S1. Number of TADs detected by three different domain-caller methods at the resolutions of 50 kb and 10 kb for Hi-C, HiChIP, and SPRITE data. Table S2. The details of the 15 TADs overlapped with heterochromatins or centromeres. Table S3. The TADs are from the overlapping between the second chromatin-state cluster and the third structural cluster in Fig. 8. The genes from the following TADs are enriched for GO terms: GO:0044278 ‘cell wall disruption in other organism’, GO:1905874 ‘regulation of postsynaptic density organization’, GO:1905606 ‘regulation of presynapse assembly’, and GO:1905606 ‘regulation of presynapse assembly’ from BPO; GO:0016342 ‘catenin complex’, GO:0099061 ‘integral component of postsynaptic density membrane’, and GO:0005913 ‘cell-cell adherens junction’ from CCO; GO:0005004 ‘GPI-linked ephrin receptor activity’, GO:0005003 ‘ephrin receptor activity’, and GO:0030594 ‘neurotransmitter receptor activity’ from MFO. The overrepresentation test was generated with PANTHER. Table S4. The TADs are from the overlapping between the twelfth chromatin-state cluster and the third structural cluster in Fig. 8. The genes from the following TADs are enriched for GO terms: GO:0050911 ‘detection of chemical stimulus involved in sensory perception of smell’, GO:0050907 ‘detection of chemical stimulus involved in sensory perception’, and GO:0007608 ‘sensory perception of smell’ from BPO; GO:0005886 ‘plasma membrane’ and GO:0016021 ‘integral component of membrane’ from CCO; and GO:0005549 ‘odorant binding’, GO:0004984 ‘olfactory receptor activity’, and GO:0004930 ‘G protein-coupled receptor activity’ from MFO. The overrepresentation test was generated with PANTHER. (PDF 332 kb) [file 12864_2019_5551_MOESM1_ESM.pdf]

TADKB: Family classification and a knowledge base of topologically associating domains

Supplementary materials

Exponent parameter

The exponent parameter of Hi-C contact matrices of TADs is calculated as follows:

(1) calculating the cumulative Hi-C contacts between any two successive bead separations determined by two successive numbers in the set of 2, 3, 4, 6, 9, 14, 22, 35, 56, 91, 149, and 247 (e.g., for the bead separation by 6 and 9, we sum all entries in the Hi-C matrix with indexes $6 \leq |i - j| < 9$);

(2) the cumulative Hi-C contacts are normalized by the length of bead separations (e.g., the length between 6 and 9 is 3), and then further normalized by the sum of all length-normalized cumulative Hi-C contacts.

(3) we next calculate the natural logarithm of contact probability and fit a linear model $\log(y) = ax+b$, where y is the contact probability and x is the bead separation. The parameter a in $y=e^{ax+b}$ is the final exponent parameter, which is used to quantify the relationship between genomic distances and the natural logarithms of contact probabilities.

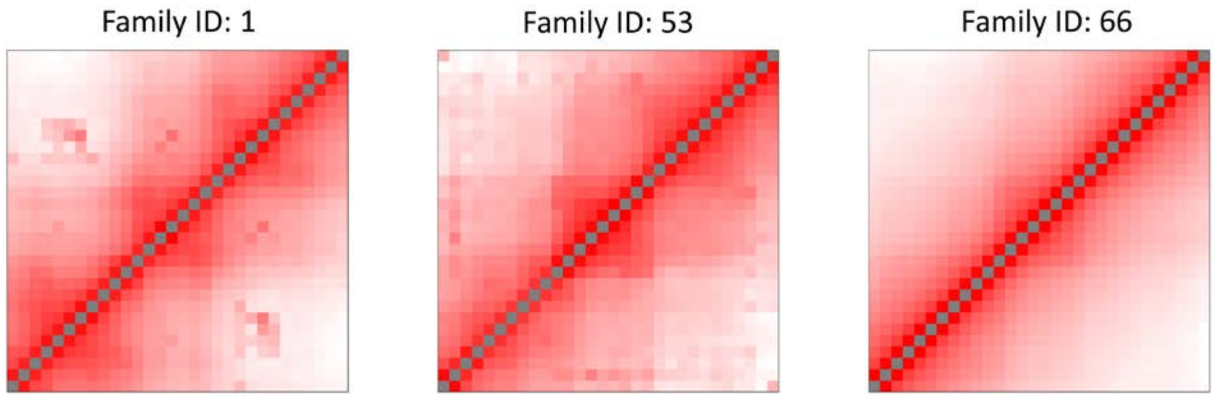

Figure S1. The 30×30 average Hi-C contact matrices calculated from all members in a TAD family. The family is from GM12878 with 20 predefined chromatin-state clusters and five predefined structural clusters.

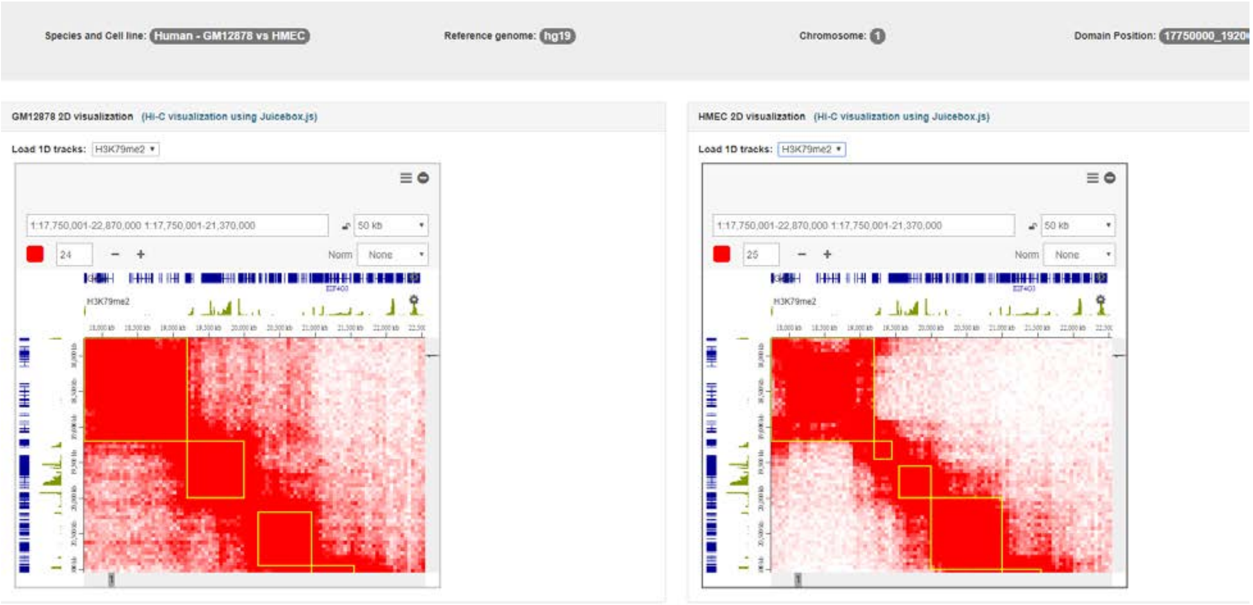

Figure S2. An example of two Hi-C heat maps for TAD pairs in acrossCells.

Table S1. Number of TADs detected by three different domain-caller methods at the resolutions of 50 kb and 10 kb for Hi-C, HiChIP, and SPRITE data.

	Cell	Resolution						Normalization	Reference
		50kb			10kb				
		DI	GMAP	IS	DI	GMAP	IS		
**Human – hg19**									
Hi-C	GM12878	2773	1754	3131	3775	2406	3301	KR	Rao et al, 2014
HiChIP	GM12878	4988	2067	3206	4296	3415	4909	ICE	Mumbach et al, 2016
SPRITE	GM12878	1789	1871	3063	2729	2451	4132	ICE	Quinodoz et al, 2018
Hi-C	HMEC	2621	1851	3154	4363	3069	4854	KR	Rao et al, 2014
Hi-C	HUVEC	2508	1730	3102	3438	2437	4346	KR	Rao et al, 2014
Hi-C	IMR90	2727	1738	3039	4107	2429	3684	KR	Rao et al, 2014
Hi-C	K562	3298	1732	3097	3828	2435	4149	KR	Rao et al, 2014
Hi-C	KBM7	2611	1755	3162	3268	2374	4409	KR	Rao et al, 2014
Hi-C	NHEK	2734	1876	3189	4213	3238	5044	KR	Rao et al, 2014
**Mouse – mm10 (CH12-LX – mm9)**									
Hi-C	ES	2075	1556	2812	3516	2105	4224	KR	Bonev et al, 2017
HiChIP	ES	2228	1624	2893	4061	2829	4436	ICE	Mumbach et al, 2016
SPRITE	ES	1626	1418	2636	2064	2084	3405	ICE	Quinodoz et al, 2018
Hi-C	NPC	1936	1522	2729	2794	1740	3681	KR	Bonev et al, 2017
Hi-C	CN	1930	1483	2660	2716	1476	3598	KR	Bonev et al, 2017
Hi-C	CH12-LX	2290	1501	2779	3204	2848	4274	KR	Rao et al, 2014

Table S2. The details of the 15 TADs overlapped with heterochromatins or centromeres.

Chromosome	TAD start	TAD end	Structural cluster ID
chr1	120800000	143550000	2
chr2	90400000	92350000	5
chr2	95300000	96500000	4
chr3	88250000	90550000	4
chr3	93500000	97450000	5
chr4	49450000	49700000	5
chr5	44800000	46450000	2
chr7	56200000	58100000	5
chr7	61500000	61950000	4
chr7	61950000	65100000	1
chr8	42250000	43850000	5
chr9	44700000	65650000	5
chr12	32900000	34900000	3
chr13	19000000	20200000	5
chr19	19650000	24650000	5

Table S3. The TADs are from the overlapping between the second chromatin-state cluster and the third structural cluster in Figure 8. The genes from the following TADs are enriched for GO terms: GO:0044278 ‘cell wall disruption in other organism’, GO:1905874 ‘regulation of postsynaptic density organization’, GO:1905606 ‘regulation of presynapse assembly’, and GO:1905606 ‘regulation of presynapse assembly’ from BPO; GO:0016342 ‘catenin complex’, GO:0099061 ‘integral component of postsynaptic density membrane’, and GO:0005913 ‘cell-cell adherens junction’ from CCO; GO:0005004 ‘GPI-linked ephrin receptor activity’, GO:0005003 ‘ephrin receptor activity’, and GO:0030594 ‘neurotransmitter receptor activity’ from MFO. The overrepresentation test was generated with PANTHER [1].

Chromosome	TAD start	TAD end	Exponent parameter	Radius of gyration
chr1	48550000	50850000	-6.93631	0.48398
chr1	187000000	190450000	-8.85921	0.53426
chr1	215850000	218400000	-7.12362	0.49203
chr1	237800000	240150000	-7.37675	0.48538
chr2	21250000	23950000	-6.94384	0.48324
chr2	50750000	53900000	-3.25223	0.41202
chr2	77100000	80150000	-1.51738	0.39378
chr2	80200000	84600000	-1.96142	0.41373
chr2	114750000	118550000	-4.65667	0.45887
chr2	124750000	127800000	-1.86766	0.41062
chr2	220550000	222850000	-2.55493	0.43414
chr3	5250000	8700000	-4.72846	0.44157
chr3	81400000	83650000	-4.5942	0.44137
chr3	88250000	90550000	-5.22286	0.47303
chr3	93500000	97450000	-4.61102	0.49394
chr3	108850000	111300000	-2.90987	0.48744
chr3	164950000	167050000	-3.19381	0.40926
chr4	27050000	30150000	-11.05662	0.52477
chr4	30350000	35950000	-5.93636	0.45574
chr4	44700000	46750000	-2.01469	0.4279
chr4	57950000	60050000	-8.87604	0.51923
chr4	61400000	65500000	-7.14377	0.48627
chr4	65550000	68250000	-3.34652	0.47318
chr4	91250000	94550000	-2.90496	0.47809
chr4	126200000	128500000	-3.35992	0.46196
chr4	134000000	137900000	-1.14683	0.34473
chr4	160400000	163150000	-3.24089	0.41931
chr4	178450000	181600000	-4.73732	0.45781
chr5	11650000	13850000	-1.29637	0.3953
chr5	18350000	21650000	-2.50522	0.36686
chr5	82950000	86050000	-2.01566	0.36017
chr5	151200000	153350000	-2.22474	0.4132
chr6	76600000	79550000	-1.13436	0.3716
chr6	91300000	93500000	-3.30413	0.4306
chr7	8450000	10950000	-6.27891	0.4907

chr7	144050000	147950000	-8.17725	0.50605
chr8	2700000	6050000	-2.28237	0.44967
chr8	13550000	17000000	-2.04847	0.40004
chr8	82850000	85500000	-2.51572	0.42533
chr8	87600000	89700000	-2.35378	0.37675
chr8	110750000	112900000	-5.96062	0.49936
chr8	136400000	139150000	-3.9493	0.42628
chr9	10000000	12500000	-3.73155	0.4202
chr9	22500000	26650000	-2.39889	0.41225
chr9	27600000	30800000	-2.58091	0.41496
chr9	104350000	107500000	-2.3974	0.40584
chr9	120350000	123200000	-5.41338	0.45139
chr10	18950000	21150000	-3.34228	0.47263
chr10	54350000	57050000	-2.25153	0.36162
chr10	65600000	69400000	-1.93868	0.37268
chr10	82950000	85850000	-1.62201	0.42062
chr10	106150000	108300000	-1.89339	0.41646
chr11	22900000	25900000	-1.97889	0.38827
chr11	39050000	41250000	-5.24437	0.46486
chr11	78300000	81250000	-6.03548	0.47228
chr11	89900000	92650000	-4.98094	0.49102
chr11	96200000	99650000	-1.46427	0.37762
chr12	16100000	18200000	-4.75825	0.51068
chr12	72350000	75000000	-3.94919	0.44155
chr12	82750000	85000000	-2.14579	0.37669
chr12	86350000	88400000	-4.44071	0.47628
chr13	53750000	58050000	-3.13623	0.41614
chr13	68000000	70050000	-2.43729	0.39751
chr13	88450000	91700000	-2.67506	0.40955
chr13	92800000	95050000	-2.4596	0.4327
chr14	25650000	29100000	-3.04472	0.40551
chr14	42250000	45350000	-1.87762	0.38821
chr14	45800000	50050000	-2.24414	0.45679
chr14	82000000	88200000	-3.25346	0.51409
chr15	86400000	89000000	-5.38531	0.46539
chr16	58800000	62200000	-2.26193	0.3823
chr16	63350000	66400000	-3.92967	0.47642
chr17	49350000	52950000	-3.98635	0.44388
chr18	25200000	27350000	-1.5641	0.38968
chr18	61650000	64550000	-3.55781	0.43204
chr18	68200000	70300000	-2.35133	0.40206
chr21	22350000	25900000	-3.83658	0.45603
chrX	27550000	30550000	-7.95821	0.51303
chrX	32350000	34400000	-6.35666	0.48998
chrX	34550000	37200000	-6.04322	0.4904
chrX	89100000	92700000	-9.60171	0.53167

chrX	93100000	99700000	-14.87602	0.56056
chrX	103600000	106050000	-6.44878	0.52107
chrX	115000000	117450000	-7.54204	0.46873
chrX	123500000	128550000	-13.14079	0.5467
chrX	144450000	146900000	-7.4326	0.50118

Table S4. The TADs are from the overlapping between the twelfth chromatin-state cluster and the third structural cluster in Figure 8. The genes from the following TADs are enriched for GO terms: GO:0050911 'detection of chemical stimulus involved in sensory perception of smell', GO:0050907 'detection of chemical stimulus involved in sensory perception', and GO:0007608 'sensory perception of smell' from BPO; GO:0005886 'plasma membrane' and GO:0016021 'integral component of membrane' from CCO; and GO:0005549 'odorant binding', GO:0004984 'olfactory receptor activity', and
GO:0004930 'G protein-coupled receptor activity' from MFO. The overrepresentation test was generated with PANTHER [1].

Chromosome	TAD start	TAD end	Exponent parameter	Radius of gyration
chr2	122550000	124750000	-4.46825	0.47297
chr4	49450000	49700000	-4.55697	0.44749
chr4	96050000	99000000	-9.75547	0.49293
chr4	187150000	191154276	-1.98385	0.40577
chr5	24600000	26700000	-1.51476	0.36907
chr6	61900000	64000000	-2.72338	0.46714
chr7	51450000	54800000	-1.55849	0.39285
chr7	61950000	65100000	-2.87523	0.46487
chr7	66800000	68850000	-3.06734	0.38637
chr8	33500000	36650000	-2.42188	0.40681
chr8	50300000	52950000	-5.05449	0.45081
chr9	66700000	67350000	-1.69291	0.41297
chr9	82800000	85800000	-2.94915	0.40362
chr11	36800000	38850000	-3.99009	0.4611
chr11	48200000	50800000	-1.6366	0.41622
chr11	54750000	57050000	-2.31918	0.45255
chr12	60350000	62600000	-4.28048	0.46185
chr12	125650000	128600000	-6.78419	0.48492
chr14	39950000	42100000	-1.64776	0.41582
chr19	27750000	29950000	-2.86113	0.40924
chr21	19650000	22250000	-2.92476	0.43112
chr22	47550000	50150000	-1.60596	0.39817
chrX	61700000	64750000	-7.45827	0.50734

Table S5. The TADs are from the overlapping between the twentieth chromatin-state cluster and the third structural cluster in Figure 8. No enriched GO terms were found in the genes from the following TADs.

Chromosome	TAD start	TAD end	Exponent parameter	Radius of gyration
chr1	79250000	81600000	-6.73559	0.48322
chr2	40000000	42050000	-2.64929	0.43344
chr2	214100000	216200000	-3.18362	0.50573
chr3	450000	3200000	-2.19739	0.41389
chr3	33900000	36750000	-1.90375	0.37204
chr3	53950000	56550000	-2.11905	0.39387
chr3	172550000	176150000	-1.65503	0.37934
chr4	117100000	119200000	-3.8505	0.42798
chr4	130250000	133650000	-2.37549	0.42239
chr5	90750000	92900000	-1.99714	0.37253
chr6	21600000	24350000	-2.25533	0.43137
chr6	114400000	116450000	-3.88263	0.45502
chr7	108250000	111400000	-3.70183	0.42232
chr7	124650000	127150000	-6.1553	0.46594
chr8	92450000	94650000	-3.11424	0.40674
chr10	108350000	111600000	-1.52288	0.38979
chr12	22850000	25000000	-2.19458	0.37604
chr13	62550000	66200000	-4.16759	0.45735
chr13	103500000	107800000	-2.49639	0.45244
chr20	58500000	60550000	-2.78692	0.44604
chrX	49350000	52150000	-4.84651	0.49082
chrX	139800000	143200000	-9.48185	0.50923

1. Mi H, Muruganujan A, Casagrande JT, Thomas PD: **Large-scale gene function analysis with the PANTHER classification system**. *Nature Protocols* 2013, **8**:1551.
